# Unsupervised pattern identification in spatial gene expression atlas reveals mouse brain regions beyond established ontology

**DOI:** 10.1073/pnas.2319804121

**Published:** 2024-09-03

**Authors:** Robert Cahill, Yu Wang, R. Patrick Xian, Alex J. Lee, Hongkui Zeng, Bin Yu, Bosiljka Tasic, Reza Abbasi-Asl

**Affiliations:** ^a^Department of Neurology, University of California, San Francisco, CA 94143; ^b^UCSF Weill Institute for Neurosciences, San Francisco, CA 94143; ^c^Department of Statistics, University of California, Berkeley, CA 94720; ^d^Allen Institute for Brain Science, Seattle, WA 98109; ^e^Department of Electrical Engineering and Computer Sciences, University of California, Berkeley, CA 94720; ^f^Department of Bioengineering and Therapeutic Sciences, University of California, San Francisco, CA 94143

**Keywords:** spatial gene expression, unsupervised learning, brain ontology

## Abstract

The rapid growth of large-scale spatial gene expression data demands efficient and reliable computational tools to extract major trends of gene expression in their native spatial context. Here, we used stability-driven unsupervised learning (i.e., staNMF) to identify principal patterns (PPs) of 3D gene expression profiles and understand spatial gene distribution and anatomical localization at the whole mouse brain level. Our subsequent spatial correlation analysis systematically compared the PPs to known anatomical regions and ontology from the Allen Mouse Brain Atlas using spatial neighborhoods. We demonstrate that our stable and spatially coherent PPs, whose linear combinations accurately approximate the spatial gene data, are highly correlated with combinations of expert-annotated brain regions. These PPs yield a brain ontology based purely on spatial gene expression. Our PP identification approach outperforms principal component analysis and typical clustering algorithms on the same task. Moreover, we show that the stable PPs reveal marked regional imbalance of brainwide genetic architecture, leading to region-specific marker genes and gene coexpression networks. Our findings highlight the advantages of stability-driven machine learning for plausible biological discovery from dense spatial gene expression data, streamlining tasks that are infeasible by conventional manual approaches.

## Significance

A comprehensive understanding of the spatial organization and differentiation of the mammalian brain requires interpreting 3D structural and molecular information in biologically plausible ways. At the moment, reliable computational methods and workflows are still lacking for reproducible analysis of gene expression data that incorporates existing domain knowledge of the brain structure. In this work, we combined stability-driven nonnegative matrix factorization with spatial correlation analysis to analyze the 3D spatial gene expression of the entire adult mouse brain. Our approach connects data-driven methods with spatial domain knowledge, revealing a gene expression-defined anatomical ontology and interpretable region-specific genetic architecture captured by the marker genes and spatial coexpression networks.

In the past decade, unsupervised explorations of large-scale single-cell transcriptomics datasets enabled by machine learning tools led to an unbiased definition of cell types—groups of cells with similar gene expression patterns (1–5). Traditionally, genetic profiling requires cell isolation that discards the spatial information of cells within tissues or organs. Spatially resolved techniques preserve the spatial information which are crucial for understanding cell function and tissue organization (6–8). Spatial patterns may correlate with specific cell types or cell type combinations and reflect local tissue characteristics in structure and function. To accommodate their growing popularity and data throughput, computational pipelines also need to incorporate spatial information in interpreting the outcome. Spatially aware analytical tools apply to both healthy and diseased tissues and may help elucidate gene and organ functions and generate viable hypotheses for disease mechanisms (9–13) in the spatial domain.

A core element of biospatial information is the anatomical atlas of an organ, which is defined by expert annotation based on accumulated historical data. Brain atlases (14, 15) are comparable across individuals and species, facilitating cross-referencing and analysis of neural data in a consistent manner. For the adult mouse brain, the Allen Common Coordinate Framework (CCFv3) is a widely used atlas and ontology (hierarchical relations between parts of the atlas) built on the Allen Mouse Brain Atlas (ABA) (16). Yet its construction is time-intensive, hard to scale, and potentially affected by human judgment. Data-driven approaches can mitigate human error, streamline the process, and uncover information hard to perceive by the human eye (17).

Currently, segmentation and clustering are the two main categories of machine learning approaches in the analysis of spatial gene expression data (17–31). While these methods yield a set of spatially nonoverlapping or, in some cases, overlapping regions, the problem formulation focuses on local information and does not explicitly model the global structure of the entire gene expression data. By contrast, matrix decomposition techniques such as non-negative matrix factorization (NMF) (32–34) provide a model-based representation of an entire dataset as a combination of a set of dictionary elements or principal patterns (PPs) (18, 35–40). These models could reduce complex spatial patterns into a combination of PPs, which provide a more interpretable representation of each data point compared to segmentation or clustering. However, the simplest matrix decomposition model, principal component analysis (PCA), despite its frequent usage (41–43), is not a sensible choice because biologically realistic assumptions, such as non-negativity, are unmet. NMF and its variants include non-negativity as an explicit constraint in the problem formulation, leading to a more biologically plausible outcome (36), with relevant applications in the analysis of gene expression (18, 35, 37, 44), neural recordings (38, 39), etc.

More importantly, we used stability-driven NMF (staNMF) algorithm (18) to incorporate stability as the central criterion in model selection to analyze spatial gene expression datasets of the adult mouse brain. Stability is a measure of scientific reproducibility and statistical robustness (45). It asks whether each step of the pipeline produces consistent results when subject to slight perturbations in the model or data (46, 47). Validating stability is also a central aspect of the veridical data science framework that introduces perturbation into the modeling pipeline as stability assessment at various stages, from data to model to inference. In scientific machine learning, stability is a minimum requirement for interpretability (45, 47) and, in the current context, essential for identifying biologically meaningful and coherent spatial patterns in the mouse brain (48). Previous work has demonstrated the promise of staNMF in interpreting 2D spatial gene expression images from *Drosophila* embryos (18). Here, we extend the analysis to 3D and, by spatial correlation analysis with an existing brain atlas (16), found that the PPs are clearly localized in single or combinations of anatomical regions, which suggests a gene expression-defined ontology beyond the one from neuroanatomy. Moreover, our analysis reveals a marked regional imbalance of gene expression, with the hippocampus having the most diverse gene expression than others, followed by the isocortex and the cerebellar regions. We recover the spatial genetic architecture using the spatial organization and correlation structure of the gene expression, which reveals region-specific marker genes as well as putative spatial gene coexpression networks (sGCNs) spanning the entire mouse brain.

## Results

### Identifying Stable PPs in the Allen Mouse Brain Atlas.

**Fig. 1.**
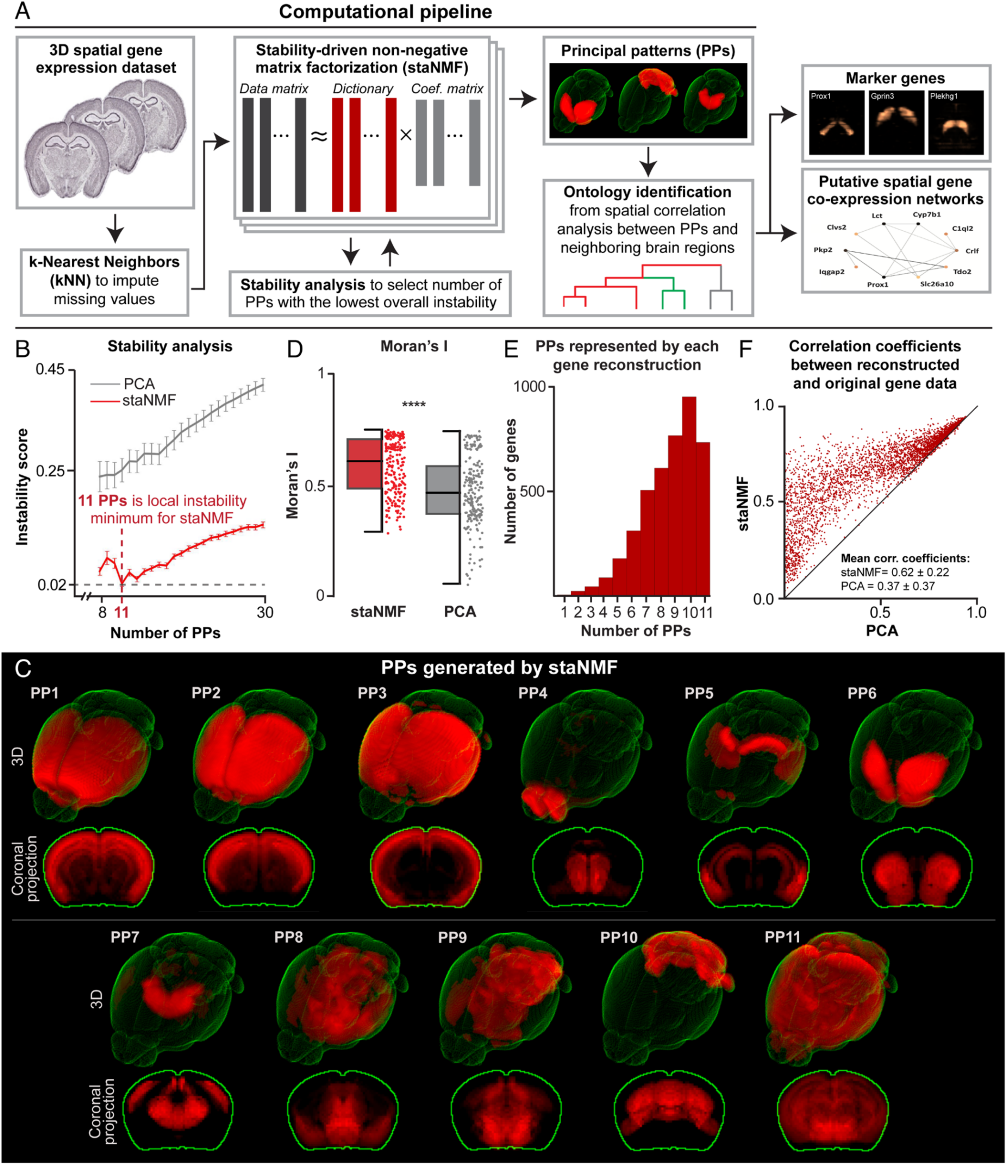
staNMF-based computational pipeline for spatial gene expression data. (*A*) Illustration of the computational pipeline with essential steps and outcomes. (*B*) Stability analysis for staNMF PPs and PCA PPs across 100 runs for each K value, from 8 to 30 for ABA dataset, using the Hungarian matching method. Error bars are the SD. (*C*) 11 PPs generated by staNMF from the ABA dataset in 3D and projected on the coronal plane. (*D*) Boxplot of Moran’s I calculated for staNMF vs. PCA PPs across 220 bootstrap simulations (*P*-value < 0.001). The data from each individual point are shown in a vertical column to the right of the boxplot. (*E*) The number of PPs represented by each staNMF gene reconstruction of the 4,345 ABA genes. (*F*) Comparison of the reconstruction accuracy between staNMF and PCA. Each dot represents one gene. The coordinates of each dot are the Pearson correlation coefficients between the measured gene expression in the ABA dataset and the reconstructed gene expression by staNMF or PCA.

We used the staNMF (13, 30) framework to extract PPs in the spatial gene expression data with additional pre- and postprocessing steps for data preparation, quality assessment, and to derive biological insights (Fig. 1*A*). PPs or the latent factors that optimally capture data variability are extracted using stability analysis to ensure the reproducibility of the PPs (referred to as stable PPs). The analysis evaluates an instability score, here defined as the average dissimilarity of all learned dictionary pairs using their cross-correlation matrix. We use the Hungarian matching method (49) (Fig. 1*B*) or an Amari-type error function (50) (*SI Appendix*, Fig. S1) to account for the invariances (*Materials and Methods*). Overall, staNMF yields two outputs: 1) *K* PPs for the whole imaging data, and 2) the coefficients or PP weights for each gene expression image. The model reconstructs each 3D gene expression profile by a non-negative linear combination of the PPs. Each PP is calculated after model training as one of *K* dictionary elements learned via staNMF. The weights of a PP or dictionary element for each gene are determined by the coefficients of staNMF. Our end-to-end pipeline is computationally efficient and can handle large datasets generated in modern spatially resolved sequencing techniques (8, 48).

We used the pipeline to determine the PPs for 4,345 3D spatial gene expression profiles in the adult mouse brain (56 d old) from the ABA dataset (48), where each gene was examined by whole-brain serial sectioning and RNA in situ hybridization (ISH) at 200 µm isotropic resolution. The preprocessing step uses a kNN-based voxel imputation to fill in approximately 10% missing voxel data in ABA. On a hold-out test set of 1,000 random voxels for each of the 4,345 genes from the ABA dataset (for 4,435,000 total hold-out data points), the mean error was smaller than 0.01. The Pearson correlation coefficient (PCC) between the measured and imputed gene expression data was 0.52, with a *P*-value < 0.01. To determine stable PPs, we calculated the instability score with 100 runs of the same algorithm across a range of 8 to 30 possible numbers of PPs. The lowest instability (and thus highest stability) was found when *K* = 11, with an instability score of 0.020 ± 0.002 (1 is the maximum instability) for the Hungarian matching method (49). The settings when *K* = 13 and *K* = 12 have the next two lowest instability scores (0.03 and 0.04, respectively, with SD < 0.01).

To assess the performance and stability of our approach, we first compared the outcome of staNMF with PCA (Fig. 1*B*). To quantify the stability of each method in identifying PPs, we performed data perturbation by bootstrapping (*Materials and Methods*). We found that staNMF PPs have higher stability and lower SD compared to PCA PPs (0.25 ± 0.01) at every value of K tested. In terms of computational runtime, staNMF takes longer to run than PCA, though both are fast-running models. On a 2021 MacBook Pro M1 laptop CPU, where the computation was tested, it takes 26 s to run staNMF to create one set of PPs on the ABA dataset vs. 4 s for PCA.

Another important aspect to evaluate is the spatial coherence of PPs, which is important for their biological interpretability. To this end, we used Moran’s I (42, 51–53), which ranges in value from –1 to 1, as a global summary statistic (Fig. 1*C*). A value close to −1 indicates little spatial organization, whereas a value close to 1 indicates a clear spatially distinct pattern. The average Moran’s I for staNMF PPs is 0.58 ± 0.12, which is considerably higher than that of PCA at 0.47 ± 0.15 (*P*-value < 0.001) across 20 bootstrap simulations for each of the 11 PPs (Fig. 1*D* and *SI Appendi*x, Fig. S2). This suggests a stronger spatial separation and coherence of PPs obtained from staNMF than those from PCA (*SI Appendi*x, Fig. S3*A* for visualization of PCA PPs). We want to point out that although staNMF PPs are spatially coherent, a large number of PPs tend to be present in most gene expression profiles (58% of all genes are represented in nine or more PPs), suggesting the spatial heterogeneity of gene expression in the adult mouse brain (Fig. 1*E*). Only two genes are represented in a single PP (<0.1% of all 4,345 genes), while 438 genes are represented in all 11 PPs (10.1% of all genes).

Additionally, we compared the staNMF and PCA reconstruction accuracy in a scatterplot (Fig. 1*F*), where each point represents one of the 4,345 genes in the dataset. We defined the reconstruction accuracy as the PCC between the reconstructed and the original gene expression image. Fig. 1*F* shows that staNMF considerably outperforms PCA in the reconstruction performance (0.62 ± 0.22 for staNMF compared to 0.37 ± 0.37 for PCA; 24% higher accuracy for staNMF). We also found that our kNN imputation of missing values improves staNMF’s reconstruction accuracy of the original dataset from 0.59 to 0.62. It is worth noting that the reconstruction accuracy slightly increases with a higher value of K (e.g., reconstruction accuracy is 0.69 for *K* = 30). However, the instability score tends to decrease significantly for higher values of K (e.g., instability score for *K* = 30 is 0.14 vs. 0.02 at *K* = 11, which is roughly 7× higher). These findings indicate that staNMF outperforms PCA in automatically generating biologically relevant patterns from spatial gene expression profiles.

### Gene Expression–Defined Ontology from Stable PPs.

**Fig. 2.**
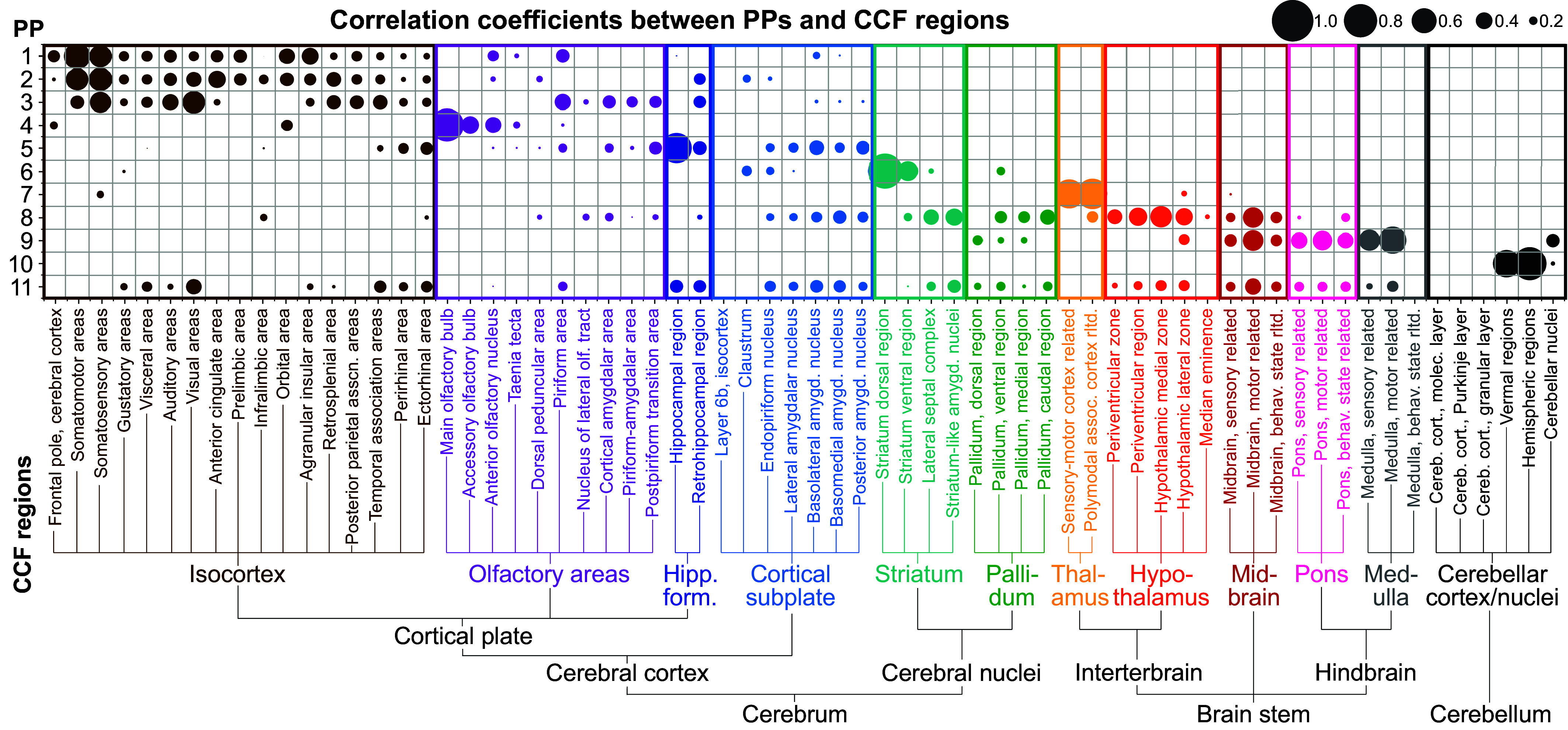
Region-dependent correlation between staNMF PPs and the CCF. Bubble plot of the Pearson correlation between PPs (y-axis) and expert-annotated regions from the CCF in the adult mouse brain (x-axis). The bubble size represents the value of the correlation coefficient between each PP and a CCF region. The CCF regions with labels are the complete set of 66 children of the 12 coarse CCF regions and are organized left-to-right based on the CCF ontology map. The PPs are organized top to bottom based on their Pearson correlation to the CCF coarse regions.

To draw connections between the PPs of gene expression and the mouse brain atlas, we investigated their overlap using spatial correlation analysis (*Materials and Methods*). Inspired by recent work on data integration in geoinformatics (54), we formulated the task as spatial entity linking between our PPs and the CCF as the knowledge base (55). We first calculated the PCC between all 868 expert-annotated brain regions (CCFv3) (16) to each of the staNMF PPs. We visualize 66 of the 868 regions in Fig. 2 to facilitate the comparison. These 66 regions provide a complete medium-level representation of the mouse brain CCF. They are selected by including all “child” regions for the 12 coarse CCF regions (isocortex, olfactory areas, hippocampal formation, cortical subplate, striatum, pallidum, thalamus, hypothalamus, midbrain, pons, medulla, and cerebellar cortex/nuclei). In this paper, we define “coarse-level” regions as these 12 CCF regions, “medium-level” regions as their 66 children, and “fine-level” regions as all regions that are finer than medium-level.

We found that the gene expression-defined PPs from staNMF have similarities, as quantified by the Pearson correlation (Fig. 2) and Dice similarity (*SI Appendi*x, Fig. S4), to the CCF ontology, but also major differences. The key signatures are consistent between the results from the two distinct similarity metrics. Three PPs (PPs 1 to 3) are well, yet in many cases, differentially correlated with selected parts of the isocortex: They all have correlations with the somatosensory areas of the isocortex, in addition to differential correlation with other cortical areas (e.g., somatomotor, visual, and orbital areas of the isocortex). Interestingly, PPs 1 to 3 also have varying representations outside of the isocortex, including in the olfactory areas, hippocampal formation, and cortical subplate, which are each viewed as part of the cerebral cortex (16). PP4 is mostly represented within the olfactory areas, especially the main olfactory bulb and orbitofrontal areas of the isocortex. PP5 has a strong correlation to hippocampal formation and, to some extent, to subregions within the isocortex, olfactory areas, and cortical subplate. Thus, we see that PPs 1 to 5 correlate with the cerebral cortex, one of the three highest-level CCF regions (in addition to the brainstem and cerebellum), but do not fit neatly within the coarse- or medium-level CCF regions.

Moving next to PP6, we found a considerably high correlation between that and the striatum with minor expression in the cortical subplate. PP7 exhibits a high correlation only to the thalamus, showing good agreement with CCF’s thalamus in the overall ontology. Unlike PP7, PP8 is spread across multiple regions, especially the hypothalamus, midbrain, striatum, pallidum, and cortical subplate (in descending correlation), which suggests that these CCF regions share gene expression patterns. Similarly, PP9 is highly correlated with multiple regions in the brainstem areas including the medulla, midbrain, and pons, as well as a minor expression in cerebellar nuclei. PP10 is highly correlated with the cerebellum, with major expression in cerebellar vermal and hemispheric regions but not in the cerebellar nuclei. A comparison between PP9 and PP10 suggests that there are significant gene expression differences between the cerebellar nuclei and the vernal/hemispheric regions of the cerebellum. Genes that are expressed in cerebellar nuclei tend to also be expressed in the brainstem areas while genes that are expressed in cerebellar vernal/hemispheric regions tend to be exclusively present in the cerebellum. PP11 is correlated to most CCF regions and visual inspection (Fig. 1*C*) suggests that it corresponds to the noisy gene expression profiles throughout the brain.

**Fig. 3.**
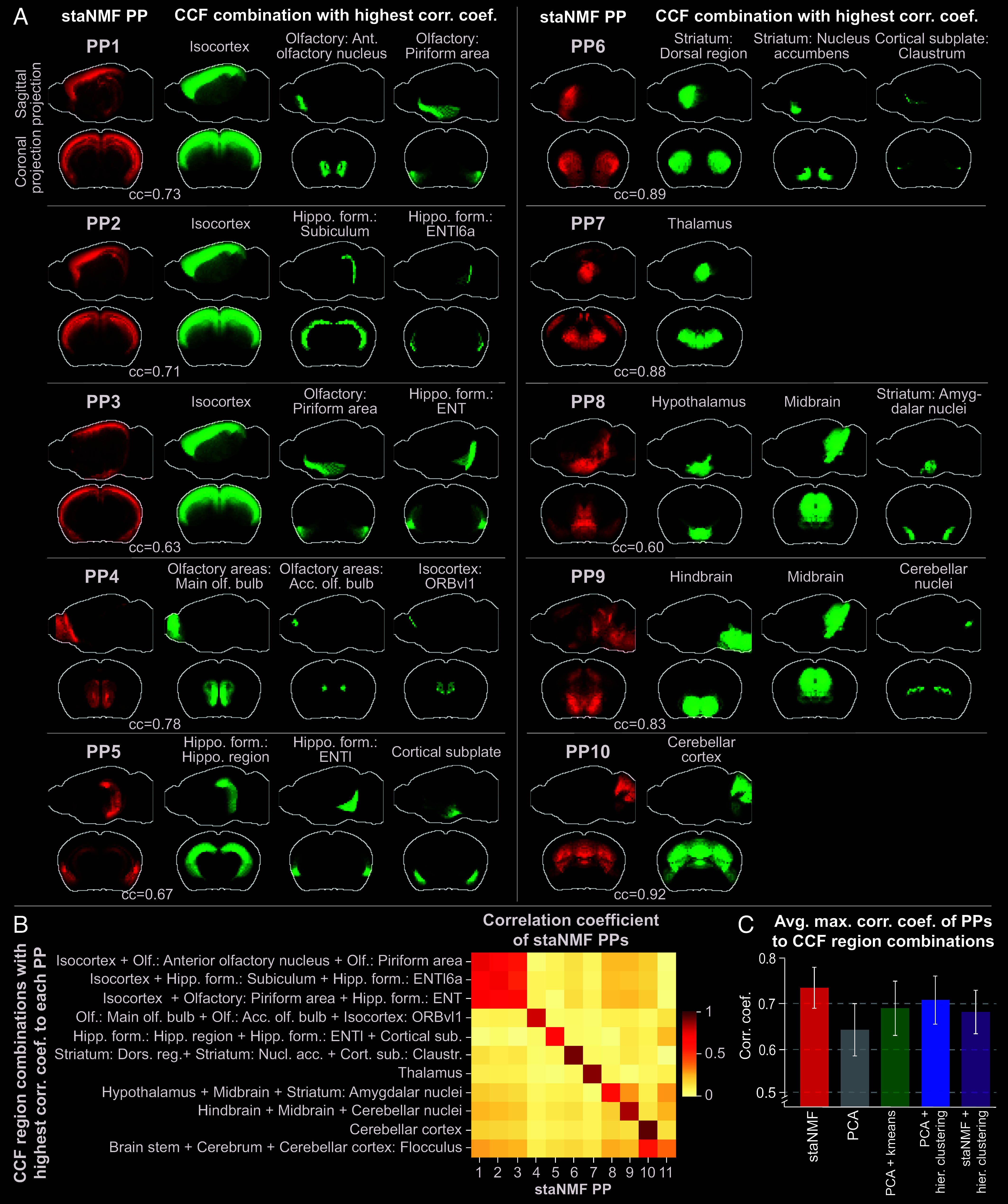
Alignment between staNMF PPs and combinations of CCF regions. (*A*) PPs (in red) and the most similar combination of expert-annotated regions (in green) from the CCFv3 (16) projected on the sagittal and coronal planes. The green regions are selected from a single or a combination of 2, or 3 neighboring regions from all 868 CCF regions with the highest PCC to each PP. The top 10 PPs are shown in descending order of the correlation coefficient. (*B*) Heatmap of the correlation coefficient between staNMF PPs and each PP’s combination of CCF regions with the highest correlation coefficient. (*C*) Comparison of the average maximum correlation coefficient of CCF region combinations to each PP for five matrix decomposition and segmentation methods: staNMF, PCA, PCA followed by k-means, PCA followed by hierarchical clustering, and staNMF followed by hierarchical clustering.

Besides examining the one-to-one relation between CCF ontology and PPs, we asked which combination of CCF regions is best aligned with each PP. To answer this question, we ran a spatial neighborhood query of combinations of 2 or 3 adjacent CCF regions (*Materials and Methods*). From a total of 868 CCF regions, we found 22,711 binary combinations and 1,834,540 ternary combinations that are spatially contiguous. We did not consider higher-order combinations due to the exponentially growing search space. We then identified the maximum PCC between each PP and the superset of all single CCF regions, all combinations of 2 CCF regions, and all combinations of 3 CCF regions. We found that the PPs tend to align with combinations of the coarse, medium, and/or fine CCF regions, but these combinations may exhibit different ontology than CCF (Fig. 3*A*):

(i) PPs 1 to 3 have their highest correlation to combinations of three CCF regions (PCC = 0.73, 0.71, and 0.63, respectively), which includes the isocortex. In addition to isocortex, PP1 adds the anterior olfactory nucleus and the olfactory piriform area, PP2 adds two finer-level retrohippocampal regions including the subiculum and the fine-level layer 6a of the lateral entorhinal area (ENTl6a), and PP3 adds the olfactory piriform area and the entorhinal area (ENT) of the retrohippocampal region.
(ii) PP4 has its highest correlation (PCC = 0.78) with the combination of olfactory bulb and accessory olfactory bulb with a fine-level cortical region (Orbital area, ventrolateral part, layer 1, referred to as ORBvl1).
(iii) PP5 is maximally correlated with a combination of two medium-level regions from hippocampal formation (hippocampal region and ENTl), and the high-level cortical subplate region.
(iv) PP6 has its highest correlation (PCC = 0.89) to a combination of three CCF regions: 1) striatum: dorsal region; 2) striatum: nucleus accumbens; and 3) striatum: olfactory tubercle. PP6 does not include the striatum: amygdalar nuclei. Instead, the combination of the hypothalamus, amygdalar nuclei, and midbrain is maximally correlated to PP8. Single-cell gene expression research has suggested that the amygdalar nucleus, midbrain, and hypothalamus contain cell types that are in fact highly related (56).
(v) PP7 and PP10 are the only PPs that are each maximally correlated with only one single CCF region: PP7 is primarily mapped to the thalamus (PCC = 0.88), while PP10 is primarily mapped to the cerebellar cortex (PCC = 0.92).
(vi) PP9 is maximally correlated with the combination of hindbrain, midbrain, and cerebellar nuclei (PCC = 0.84). It organizes the midbrain and hindbrain together, and suggests a relatively high similarity of gene expression between the midbrain, medulla, and pons, as observed with single-cell transcriptomics and clustering (56).

These observations indicate that the PPs from the spatial gene expression partition the mouse brain differently from the CCF, suggesting a distinct ontology. We verify the uniqueness of the partitions by examining the correlation matrix between all PPs and their associated CCF combinations (Fig. 3*B*). Most PPs (except PPs 1 to 3, and 11) exclusively map to their associated CCF region combinations, suggesting low overlap between these PPs. The average maximum correlation coefficient between PPs and their respective CCF region combination is 0.74 ± 0.04. By contrast, the average correlation coefficient between each PP and the CCF region combinations is 0.10 ± 0.17, except for its highest correlation region. PP11 has the lowest maximum correlation coefficient (0.37 vs. 0.60 as the next lowest) to other CCF regions, further suggesting its role in accounting for the noise in gene expression profiles. The outcome of entity linking (55) through spatial correlation analysis facilitates the construction of a gene expression-defined ontology based purely on spatial gene expression data (*SI Appendi*x, Fig. S5).

Subsequently, we conducted a similar analysis using the outcomes from common methodologies used in the segmentation and clustering of spatial gene expression data. staNMF PPs have a higher average correlation coefficient to their respective CCF regions (0.73 ± 0.05) compared to PCA PPs (0.63 ± 0.06). Furthermore, the stronger diagonal pattern in the correlation matrix for staNMF (Fig. 3*B*) compared to PCA (*SI Appendi*x, Fig. S3*B*) suggests that staNMF PPs have a better alignment with the annotated brain regions. Additionally, we conducted the same spatial correlation analysis on PPs from typical clustering techniques. We clustered the ABA dataset using 1) PCA followed by k-means clustering [similar to the stLearn framework (57)], 2) PCA followed by agglomerative hierarchical clustering [similar to the AGEA framework (17)], and 3) staNMF followed by hierarchical clustering as a point of comparison (Fig. 3*C*). staNMF has the most similar PPs to their optimal CCF regions (PCC = 0.73±0.05), whereas PCA, PCA followed by k-means, and PCA followed by hierarchical clustering produce PPs less resembling CCF regions (PCC = 0.63 ± 0.06, 0.68 ± 0.06, and 0.70 ± 0.06, respectively). Additionally, staNMF PPs have a higher similarity to CCF region combinations compared to staNMF followed by hierarchical clustering (PCC = 0.67 ± 0.05). The comparison demonstrates that the PPs from staNMF alone are more similar to the combinations of known brain regions compared to PCA or standard clustering approaches.

### Substructures of the Mouse Isocortex in PPs.

**Fig. 4.**
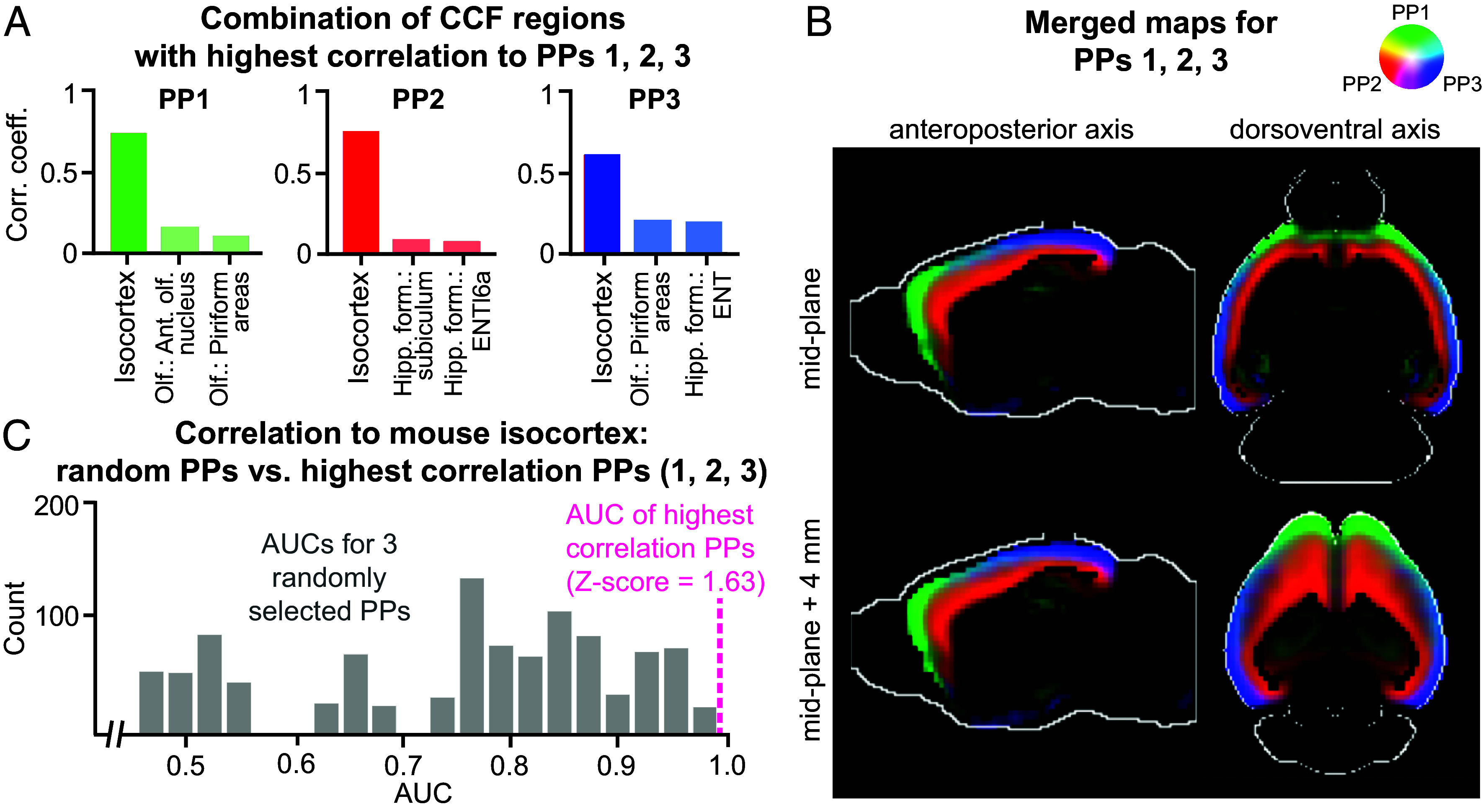
The defining PPs for the mouse isocortex. (*A*) The three CCF regions with the highest correlations with PPs 1 to 3, with the isocortex being the most correlated region. (*B*) Merged map of the PPs 1 to 3 in the isocortex. Each image is a 2D cross-section viewed along the anteroposterior or dorsoventral axis (two columns). The rows represent the cross-section used in visualization. (*C*) Histograms of AUC values for isocortex for 1,000 runs of a logistic regression randomly fitting three PPs to isocortex CCF regions. The magenta vertical dashed line indicates that the AUC for PPs 1 to 3 are the best predictors of the isocortex compared to any other three random PPs.

The mouse isocortex is a layered structure (58) with gene expression gradients along the anteroposterior and mediolateral axes (5). This information, subject to the limit of data resolution, is also reflected in the PPs 1 to 3. We observe that for each of these PPs, the correlation coefficient to the isocortex dominates that to the other regions (Fig. 4*A*). For example, PP2 has a correlation coefficient of 0.70 to the isocortex, but only 0.13 and 0.08 to other two regions that make up its highest correlated combination. Similarly, PPs 2 and 3 have correlation coefficients of 0.70 and 0.54 to the isocortex, respectively, while their correlation coefficients to other regions are considerably lower (Fig. 4*A*). Visualization of these three PPs suggests that they represent different spatial regions of the isocortex, in addition to minor components of the hippocampus and olfactory areas (Fig. 4*B*). Anatomically, PP1 represents the superficial layers in the frontal areas of the cortex, in addition to a partial representation of the anterior olfactory nucleus and the piriform area of the olfactory areas. PP2 represents the deeper layers of the isocortex in dorsolateral regions and has a minor correlation to the subiculum and entorhinal area (lateral part, layer 6a) within the retrohippocampal region. PP3 represents the superficial layers of the isocortex in dorsal regions as well as the piriform area of the olfactory areas and the entorhinal area of the retrohippocampal region. PP1 and PP3 have a gradual overlap in superficial layers, as indicated by the cyan color in Fig. 4*B*.

We investigated how effectively the combination of PPs 1 to 3 can recreate the isocortex alone by training a logistic regression model to predict the isocortex CCF reference map from PPs 1 to 3. The area under the receiver operating characteristic (ROC) curve or AUC measure for the prediction is 0.99. The regression model is the most accurate model among 1,000 other models that uses a random selection of three PPs to predict isocortex (Fig. 4*C*). The median AUC for these 1,000 models is 0.78 (compared to 0.99 for the model that uses PPs 1 to 3, as shown in the magenta vertical dashed line), demonstrating that they represent the isocortex as a whole.

### From PPs to Marker Genes.

**Fig. 5.**
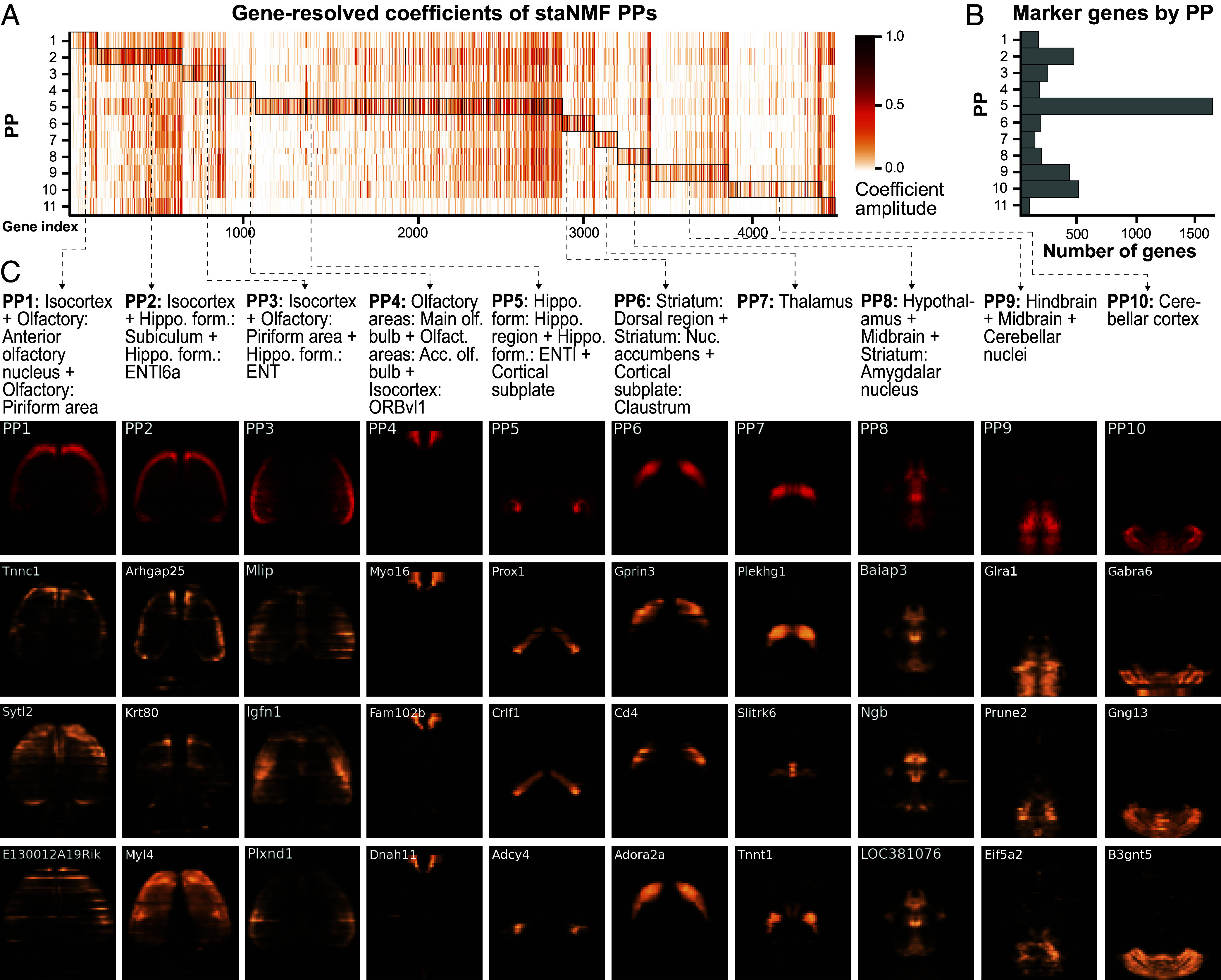
Marker gene identification from PPs. (*A*) Heatmap of the gene-resolved coefficients of the 11 PPs, in descending order by the maximum correlation coefficient to a combination of CCF regions, as described in Fig. 3. Genes are assigned to the PP for which they have the highest coefficients. (*B*) The number of significant genes for each PP, which is counted from the highlighted regions (by rectangular boxes) in *A*. (*C*) The horizontal projection of the respective PP (in red) and the corresponding gene expression of the top three marker genes (in copper). The respective CCF region combinations are provided as text above. The name of the marker gene is displayed in the top left corner of each horizontal view of gene expression.

Marker genes for an organ or tissue region are a set of genes with high expression within that region and relatively low expression in other regions. These genes are frequently used as starting points for understanding functions of cells and their local organization and to design genetic tools for experimental access to those cell types and regions for further knockout studies (59, 60). Given the relationship between PPs and brain regions established previously, one can robustly identify region-specific marker genes using the contributions of genes to the PPs. We visualize in Fig. 5*A* the gene-resolved coefficients ($a_{\mathrm{kj}}$) for each PP, where the genes are first ordered by the PP with the highest coefficient and then by their corresponding importance scores, $r_{j}=a_{\mathrm{kj}}/\sum_{j}a_{\mathrm{kj}}\;\left(k=1,2,\ldots ,K\right)$. The total number of genes selected by the PPs is not uniform across the board (Fig. 5*B*). Noticeably, PP5 (correlated with the hippocampal region) has by far the most unique genes, with over 1,500 genes. PP2 (correlated with the isocortex), PP9 (correlated with the hindbrain), and PP10 (correlated with the cerebellar cortex) also have an especially large number of associated genes (represented by darker orange and red in the heatmap).

Drawing from these observations and the previous work on *Drosophila* embryos (18), we used the following procedure to identify the marker genes for each PP: We first extracted the staNMF coefficients, ${\{a}_{\mathrm{kj}}\}\left(k=1,2,\ldots ,K\right)$, for the *j*th gene. Each gene-resolved coefficient quantifies the contribution of the kth PP in explaining the expression of the jth gene. We then assigned each gene to a specific PP with the highest coefficient. Next, we calculated the importance score $r_{j}$ for the *j*th gene to obtain the PP-level marker genes. The top three genes with the highest importance scores for each PP are visualized in Fig. 5*C*, which shows convincing visual alignment with the corresponding brain regions. More importantly, we found that the regional designation of marker genes in the mouse brain has biological relevance. For example, *Prox1*, the top-ranked marker gene obtained using the procedure for PP5 (associated with hippocampal formation and cortical subplate), is known to be widely expressed across the brain during development, but primarily in the hippocampus and cerebellum in adulthood (61). As another example, *Gabra6*, the top-ranked marker gene for PP10 (associated with the cerebellar cortex) is known to be preferentially expressed in the cerebellum as part of a program related to differentiation (62). Furthermore, we compared the PP-level marker genes found using the ranking procedure described here with the cell type-specific marker genes in the same region from a more recent scRNA-seq dataset (56). The results, summarized in *SI Appendi*x, Table S1, confirm that despite the different spatial resolution and sequencing measurements, there exists a significant overlap between the region-specific marker genes. The single-cell level measurements also provide complementary information on the cell type identities to the PPs.

### From PPs to sGCNs.

It is known that the spatial coexpression of genes yields meaningful biological relationships (63, 64). For example, an sGCN has successfully reconstructed the gap gene regulatory network in *Drosophila* (18). However, few existing computational tools incorporate spatial information in identifying gene coexpression networks, and the ones that do only leverage existing expert-defined ontologies (65–68). Data-driven ontologies from tools like staNMF will allow better identification and exploration of 3D spatial gene networks.

**Fig. 6.**
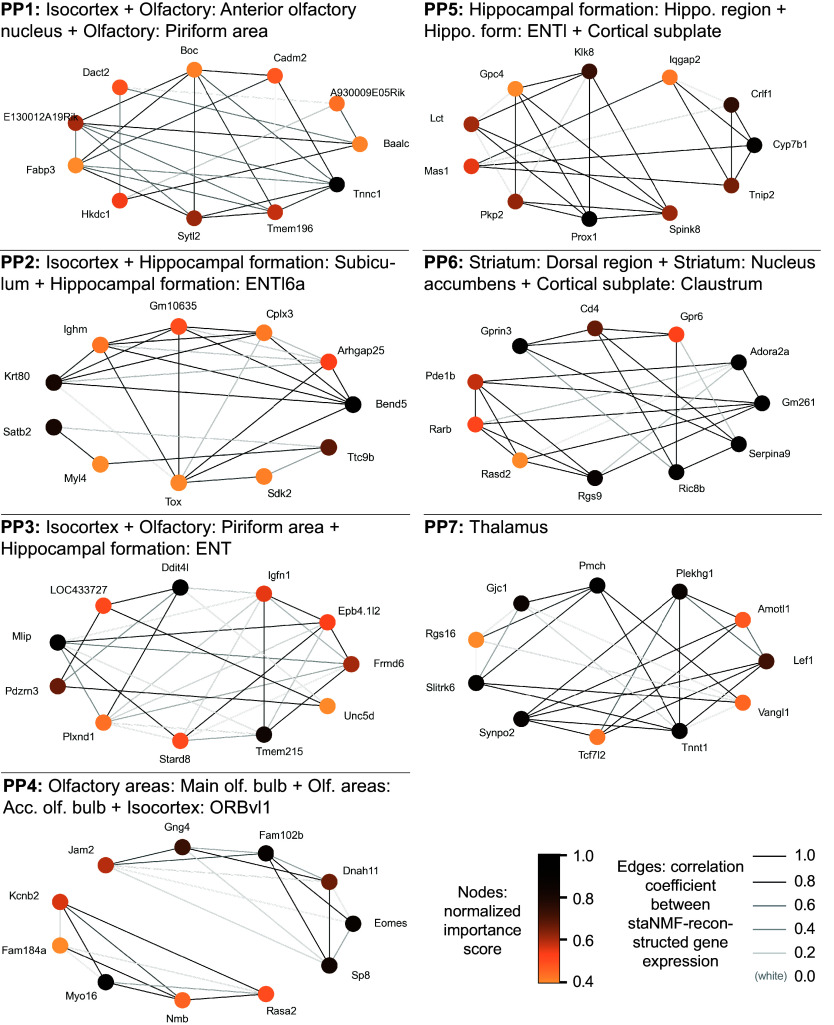
Putative spatial gene coexpression network (sGCN) construction. The sGCNs from PPs 1 to 7 and their associated brain regions from the CCFv3 are shown. The node color presents the selectivity of the gene to the PP associated with the brain region. An edge is drawn between genes if the similarity score is among the top 5% of all similarity scores for that gene subset. The edge color is proportional to the Pearson correlation of the reconstructed gene expression images of the two coexpressed genes.

Building on a similar analysis for *Drosophila* embryos (18), we used a similar procedure to construct putative sGCNs for the PPs in the adult mouse brain. Our analysis selected 10 or 11 top-ranked genes for each PP to construct the putative sGCNs (Fig. 6 for PPs 1 to 7, and *SI Appendi*x, Fig. S6 for the remaining PPs). Interestingly, some of the regulatory relationships that are recently found via experimental research are present in the sGCNs. For example, in PP6, which is correlated to the striatum, seven selected genes show especially strong edges (*Gprin3*, *CD4*, *Gpr6*, *Ric8b*, *Rgs9*, *Serpina9,* and *Gm261*) and seem to form a hub of connections. Interestingly, a 2019 experimental study in mice found that *Gprin3* controls striatal neuronal phenotypes including excitability and morphology, as well as behaviors dependent on the striatal indirect pathway and mediates G-protein-coupled receptor (GPCR) signaling (69). *Gpr6* is a GPCR gene, and *Rgs9* and *Ric8b* are regulators of GPCR genes. In addition, *Gm261* and *Serpina9* are known to impact synapse development. In addition, *Prox1* and *PKP2* appear as interactions in PP5, which is related to hippocampal formation. Interestingly, a recently published experimental study has identified *Prox1* as a transcription factor associated with *PKP2* expression (70). These relationships could be used as leads for experimental validation when studying specific genes in their tissue context.

## Discussion

Unsupervised matrix factorization models are powerful machine learning tools for exploratory data analysis in spatial transcriptomics. Combining accurate unsupervised models with stable learning improves the interpretability of the resulting spatial patterns (i.e., PPs), as we have shown using staNMF in the present work. Our pipeline can automatically find consistent gene expression-defined spatial regions in 3D without supervision, eliminating the need for manual annotation. We point out here that anatomical atlases such as the ABA (16) are constructed by dividing the brain volume into spatially contiguous regions without overlap. However, at the tissue or cellular level, this idealization is not always satisfied and the strict division should be regarded as an approximation. Gene expression-defined PPs provide an automated way to explore whole-brain data with simplistic, spatially coherent regions that retain meaningful connections to the expert-annotated anatomical atlas.

Despite the limited spatial resolution, our analysis of the current dataset encompassing the entire adult mouse brain reveals promising marker genes with region specificity for future investigations in controlled experiments. As biological processes occur in 3D space and time, analysis of the coexpression network is inherently more reliable with data from 3D gene expression. The specific sets of genes and their spatial coexpression that contribute to PPs are also likely to contribute to the unique functions of brain regions they delineate. Those genes or their combinations identified through spatial correlation analysis will be highly informative for designing genetic toolkits to experimentally access specific cell types and spatial domains within the organ of interest (71, 72). When combined with developmental data, these biological insights may help understand the longitudinal evolution of region-dependent gene expression to uncover signatures and functions hard to decipher from traditional transcriptomic methods without spatial information. Although the putative sGCNs identified here still need to be validated in controlled experiments, they may be linked to regulatory interactions, such as hub genes which are likely to mediate communication between networks, and to relationships between genes and gene modules. Our data-driven gene network representation might also be useful for studying disease processes such as selective vulnerability of certain regions to spread of pathogenic proteins in the brain (73). Through integration with scRNA-seq datasets (56), these networks can be used to study the cell-type specificity of spatial interactions between genes and find cell-type-specific gene networks. The computational pipeline in the present work leverages the linear relationship between PPs to identify gene networks. Future work could incorporate nonlinear interactions using supervised methods such as iterative random forest (74) to uncover complex gene interactions at the scale of the mouse brain.

Moreover, the availability of many different modalities for whole-organ imaging (6, 7) highlights the need for computational method developments along this direction. These methods would not only avoid human labor but are also more likely to be informative for investigating the functions of these regions. Besides gene expression data, the staNMF is also applicable to a broad range of biological data and may be used in multimodal data integration by combining learned representations. Potential future work will include integration with other modalities such as MRI and axonal projections to precisely characterize finer brain regions (58, 75). The computational efficiency of staNMF can be further improved to accommodate large datasets by exploiting the block structure of the data matrix or to use hierarchical updating schemes. The spatial neighborhood query in our computational pipeline may be upgraded into a discrete tree search to accommodate the existing brain ontology to explore higher-order combinations of brain regions. It is worthwhile to incorporate similar stability analysis in existing region-constrained matrix factorization models (39, 40) to assess the changes in the outcome. We are hopeful that the three principles for data science: predictability, computability, and stability (PCS) (47) for veridical data science, as illustrated here, will be implemented in more case studies to improve the reproducibility of data-driven scientific discovery.

## Materials and Methods

### Data Description and Preprocessing.

The primary dataset used in our study is the ISH measurements from 4,345 genes at 200 µm isotropic resolution (a matrix size of 67 × 41 × 58 for each gene expression image) from the adult mouse brain at 56 d postnatal (48). The data were collected at the Allen Institute for Brain Science and are publicly available under the Allen Brain Atlas (ABA) (https://mouse.brain-map.org/), as previously described (48). An API enables the download of the data at http://help.brain-map.org/display/mousebrain/API. The Allen Mouse Brain Common Coordinate Framework (CCF) was used as the 3D reference atlas (16). We used CCFv3 publicly available at http://help.brain-map.org/display/mousebrain/api, which consists of parcellations of the entire mouse brain in 3D and at 10 μm voxel resolution. The CCF provides labeling for every voxel with a brain structure spanning 43 isocortical areas and their layers, 329 subcortical gray matter structures, 81 fiber tracts, and 8 ventricular structures. The methods for constructing the CCF dataset are previously described in detail (16).

During preprocessing, we imputed missing voxels in the gene expression data using a k-nearest neighbors algorithm (76) with six neighbors. To test the efficacy, we calculated the accuracy on a hold-out test set of 1,000 random voxels for each of the 4,345 genes from the ABA dataset (for a total of 4,435,000 data points). Following data imputation, we created a brain mask representing all the voxels of the mouse brain using the CCF which results in 55,954 voxels, vs. the total cube array of 159,326 voxels, reducing the number of voxels used for subsequent analysis by roughly two-thirds. Once the analysis was run, we unmasked the analysis outcomes and transformed the data back to the original shape (67 × 41 × 58). The data processing uses the codebase *osNMF* (https://github.com/abbasilab/osNMF), short for ontology discovery via staNMF.

### The staNMF Framework.

NMF (32–34) decomposes the data matrix into K dictionary elements and associated coefficients, resulting in parts-based representations of the original data. Stability-driven NMF (staNMF) (18) is a model selection method that helps determine K through stability analysis. Here, we apply staNMF to the 3D gene expression data collected in the adult mouse brain as a key step in the computational pipeline (Fig. 1*A*). Following the staNMF processing pipeline, we first transformed the imputed data into a matrix of voxels by genes (of size 55,954 by 4,345). The voxels were then masked to leave out only those in the brain as previously described. The voxel-by-gene matrix is the input of the NMF algorithm, which factorizes the gene data matrix into PPs. Formally, let $X=\left[x_{1},x_{2}{,...,x}_{v}\right]$, be a v×n data matrix, where v is the number of unique voxels and n is the number of genes represented. Let $D=\left[d_{1},d_{2}{,...,d}_{K}\right]$, be a v×K matrix, representing a dictionary with K elements or atoms (columns of D), and $A=\left[a_{1},a{,...,a}_{n}\right]$, be a K×n matrix, representing the coefficient matrix. Under the current problem setting, NMF aims to minimize the loss function

$$L_{\text{NMF}}=∥X-DA{∥}_{F}^{2},$$

subjecting to non-negativity conditions D≥0, A≥0. The subscript F indicates the Frobenius norm. We used the *scikit-learn* (77) implementation of NMF with default settings of the tolerance of the stopping condition (tol = 0.0001) and the maximum number of iterations (max_iter = 200). The staNMF is trained using coordinate descent (solver = “cd”), which alternately optimizes the D and A matrices and is frequently used for NMF (78).

The stability analysis for NMF selects the parameter K computationally using an instability score. The NMF implementation used in the prior work (18) adopted an online learning algorithm, which merged the perturbations on the initialization and the data. The *scikit-learn* implementation of NMF decouples the two steps and uses a stable initialization method (79). Therefore, we used a fixed initial condition and performed data bootstrap for stability analysis. Specifically, we ran the NMF algorithm *N* = 100 times at each integer value of K from 8 to 30. Each run uses a different random seed to bootstrap sample the data. For each K, we compute an instability score that is the dissimilarity of learned dictionary pairs (**D** and **D**′) averaged over N runs. According to this definition, the optimal choice of K would result in highly stable dictionaries by data bootstrap. The dissimilarity (dsim) is formulated using the cross-correlation (xcorr) matrix, $C=xcorr\left(D,D^{\prime }\right)$, between each dictionary pair and requires accounting for the scaling and permutation invariance of the learned dictionary elements (33, 80). Cross-correlation directly accounts for the scaling invariance between dictionaries in its normalization factor. To account for permutation invariance, we chose two distinct ways: The first way is to solve an assignment problem for the columns of **D** and **D**′ beforehand using the Hungarian matching (HM) method (49), followed by calculation of the dissimilarity score,

$$\mathrm{dsim}\left(D,D^{\prime }\right)=\frac{1}{K}\sum_{1\leq \left(k,l\right)\leq K}\left(1-C_{\mathrm{kl}}^{\mathrm{HM}}\right),$$

where $\left(k,l\right)$ indicates assigned index pairs of **D** and **D**′ (K index pairs in total), and the HM superscript indicates the cross-correlation matrix **C**’ calculated after applying the HM method. The second way is to account for the permutation invariance directly in the formulation of the dissimilarity metric using an Amari-type error function (50),

$$\mathrm{dsim}\left(D,D^{\prime }\right)=\frac{1}{2K}\left(2K-\sum_{l=1}^{K}\underset{1\leq k\leq K}{\text{max}}C_{\mathrm{kl}}-\sum_{k=1}^{K}\underset{1\leq l\leq K}{\text{max}}C_{\mathrm{kl}}\right).$$

In either definition, the dissimilarity metrics are aggregated into the instability score γ using a simple average over $N\left(N-1\right)/2$ distinct pairs (18),

$$\gamma \left(K\right)=\frac{2}{N\left(N-1\right)}\sum_{1\leq p<q\leq N}\mathrm{dsim}\left(D_{p},D_{q}\right).$$

Stability analysis using either construction of the instability score yields the same result (K=11) for the most stable number of PPs (Fig. 1*B* and *SI Appendi*x, Fig. S1). While γ is on the order of 10^−2^ around the optimal value of K when performing data bootstrap only, it is at the level of 10^−9^ to 10^−10^ when using random initialization only. This sanity check shows that the instability from random initialization is negligible compared with data bootstrap.

### Spatial Neighborhood Query.

A brain atlas or parcellation B, with dimensions a×b×c, is a set of connected volumes, also called brain regions or parcels (15), ${\left\{B_{i}\right\}}_{i=1}^{n}$ such that ${⋃}_{i=1}^{n}B_{i}=B$. Numerically, each $B_{i}$ is represented by a 3D segmentation mask, $B_{i}\in R^{a\times b\times c}$, where the voxels within the mask (i.e., the support) have the value of i and those outside are 0. For the CCFv3 ontology (16) used in this work, n=868. The brain atlas is organized hierarchically based on biological knowledge of the brain regions, however, their precise spatial relationships are not explicitly given. We construct an adjacency list representation of the spatial relationship between brain regions for the subsequent analysis. This representation is commonly used in the spatial computing (81) and image processing (82) communities for its convenience. We call two brain regions, $B_{i}$ and $B_{j}$, neighboring or spatially contiguous if they contain adjacent voxels. Because the support of each $B_{i}$ has a different shape, we carried out the spatial queries of neighboring brain regions using image morphological (i.e., binary dilation) and logical operations to obtain the adjacency list. A pseudocode for generating all pairwise neighbors $\left\{\left(B_{i},B_{j}\right)\right\}$ of brain regions is given in *SI Appendix*, Algorithm 1. The triplewise neighbors $\left\{\left(B_{i},B_{j},B_{l}\right)\right\}$ are generated similarly starting from existing pairwise neighbors, while the condition for spatial contiguity is that the third region $\left(B_{l}\right)$ after dilation is overlapping with at least one member ($B_{i}$ or $B_{j}$) of a neighboring pair.

### Spatial Correlation Analysis and Entity Linking.

Entity linking is the task of connecting entity instances to an existing knowledge base for the purpose of data integration (55). Although the task traditionally concerns only entities in text data, it is increasingly referring to similar problem settings encountered in multidimensional and multimodal data (83). In geoinformatics, spatial entity linking has been widely used in integrating data with multiple features such as name, location, shape, type, etc. (54). In a similar vein, in the current work, the brain regions defined in Allen CCFv3 contain information of their anatomical name, shape, and location, while the latent factors, i.e., PPs from staNMF, contain only the shape and location information. We choose to use spatial correlation analysis to link the PPs with known anatomical regions in the Allen CCFv3 to uncover their relations.

The overlap between the PPs and the brain regions is calculated by Pearson correlation (Corr). Let $X_{k}$ be the volumes defined by a PP with index k, our spatial correlation analysis seeks the combination of spatially contiguous regions that maximize the Pearson correlation. For the k th PP, the expression for maximal correlation with two $\left(\rho_{k,\left(2\right)}^{*}\right)$ and three $\left(\rho_{k,\left(3\right)}^{*}\right)$ regions for are written as

$$\rho_{k,\left(2\right)}^{*}=\text{max Corr}\left(X_{k}\left(s\right),{\tilde{B}}_{\left(2\right)}\left(s\right)\right)=\text{max Corr}\left(X_{k}\left(s\right),{\tilde{B}}_{i}\cup {\tilde{B}}_{j}\right),$$

$$\rho_{k,\left(3\right)}^{*}=\text{max Corr}\left(X_{k}\left(s\right),{\tilde{B}}_{\left(3\right)}\left(s\right)\right)=\text{max Corr}\left(X_{k}\left(s\right),{\tilde{B}}_{i}\cup {\tilde{B}}_{j}\cup {\tilde{B}}_{l}\right),$$

where ${\tilde{B}}_{i}\cup {\tilde{B}}_{j}$ denotes the combined region of the pair $\left(B_{i},B_{j}\right)$ after mask normalization (${\tilde{B}}_{i}=B_{i}/i$). Similarly, ${\tilde{B}}_{i}\cup {\tilde{B}}_{j}\cup {\tilde{B}}_{l}$ denotes the combined region of the triple $\left(B_{i},B_{j},B_{l}\right)$ after mask normalization. The terms ${\tilde{B}}_{\left(2\right)}\left(s\right)={\tilde{B}}_{i}\cup {\tilde{B}}_{j}$ and ${\tilde{B}}_{\left(3\right)}\left(s\right)={\tilde{B}}_{i}\cup {\tilde{B}}_{j}\cup {\tilde{B}}_{l}$ may be regarded as random variables indicating the random combinations of regions, where s denotes the spatial coordinates. The maximization is conducted by exhaustive search over the respective adjacency list obtained from the spatial neighborhood search.

### sGCNs.

The putative sGCNs were constructed at the PP level. We first identified the top-ranked genes for each PP by selecting the genes with the top 0.25% importance scores ($r_{j}$) correspondingly. The selection captures all the prominent coexpression patterns in the gene expression data. This step yields a PP-specific gene subset, and these genes form the nodes of each sGCN. The edges of the network are determined by the level of correlation between the nodes. We then computed the PCC between the reconstructed 3D gene expression images (including those shown in Fig. 5*C*) for the selected genes within each subset. An edge is drawn between two genes if their correlation coefficient is among the top 5% of all coefficients of that gene subset.

## Supplementary Material

Appendix 01 (PDF)

## Data Availability

The code and intermediate files are freely available at https://github.com/abbasilab/osNMF (84). The data used in this study is publicly available under the Allen Brain Atlas (ABA) (https://mouse.brain-map.org) (16, 85).
