# Supplementary material for: Unsupervised pattern identification in spatial gene expression atlas reveals mouse brain regions beyond established ontology
Source: Proc Natl Acad Sci U S A. 2024 Sep 3;121(37):e2319804121. doi: 10.1073/pnas.2319804121 (PMC11406299; doi:10.1073/pnas.2319804121)
Supplement: Supplementary file 1 — Appendix 01 (PDF) [file pnas.2319804121.sapp.pdf]

PNAS

Supporting Information for

Unsupervised pattern discovery in spatial gene expression atlas reveals mouse brain regions beyond established ontology

Robert Cahill[1,2,#], Yu Wang[3,#], R. Patrick Xian[1,2], Alex J. Lee[1,2], Hongkui Zeng[4], Bin Yu[3], Bosiljka Tasic[4], Reza Abbasi-Asl[1,2,*]

[1]University of California, San Francisco
[2]Weill Institute for Neurosciences
[3]University of California, Berkeley
[4]Allen Institute for Brain Science

[#]indicates equal contribution
*Corresponding author: Reza Abbasi-Asl (Reza.AbbasiAsl@ucsf.edu)

This PDF file includes:

- Supporting Information
- Figures S1 to S6
- Table S1

SI-1. Moran's I

To quantify the spatial coherence of PPs, we used Moran's I statistic (1). It was originally used in geostatistics and has more recently been used in spatial gene expression literature (2). Moran's I ranges in value from -1 to 1. A value close to -1 indicates little spatial organization, similar to a chess board with black and white squares distributed across the board. A value close to 1 indicates a clear spatially distinct pattern, such as if all the black squares in a chess board were on one side and all white squares on the other. We calculated Moran's I as follows (2):

$$I = \frac{N}{W}\frac{\sum_{i=1}^{N}\sum_{j=1}^{N} w_{ij}(x_i - \underline{x})(x_j - \underline{x})}{\sum_{i=1}^{N}(x_i - \underline{x})^2}.$$

Here, x_i and x_j represent the PP coefficient at voxel locations *i* and *j*, respectively. $\underline{x}$ is the mean gene expression level of each PP. *N* is the total number of voxel locations, w_{ij} is the spatial adjacency relationship (based on the adjacency matrix, *w*) between voxels *i* and *j*. *W* is the sum of all entries in *w*, which represents the cumulative total adjacencies. We mask the dataset to only include the brain region. Then, for each voxel, we select up to 6 voxels for determining adjacency (up, down, left, right, forward, background, where available), following the "rook" definition of neighborhood. We assign w_{ij}=1 if voxel *j* is adjacent to i, and w_{ij}=0 otherwise. Given the large size of the adjacency matrix (159,326 x 159,326), we downsampled the PPs by removing every other row in each of the three dimensions to improve computational efficiency. Given certain voxels had multiple PPs with small but non-zero coefficients, we assigned each voxel in the brain map to the PP with the highest coefficient for that voxel. This ensures that unique voxels are not represented by multiple PPs.

SI-2. 3D visualizations of PPs

The 3D gene visualizations were performed using Napari viewer, a multi-dimensional image viewer for Python (3). Key settings in Napari for PPs included: opacity=1, gamma=1, blending='additive', depiction='volume', and rendering='attenuated MIP'. MIP stands for maximum intensity projection, which enhances the 3D representation of objects. We moved the slide bar to 20% from the left side for 'attenuated MIP.'

SI-3. Algorithm 1: Spatial neighborhood query (pairwise in 3D)

Algorithm 1 Spatial neighborhood query (pairwise in 3D)

```
function PairwiseNeighbors (atlas)
    # Calculate the number of brain regions
    n = Unique (atlas)
    adjacency_list = []
    all pairs = Combinations (Range (1,n))
    # Check the overlap between each pair
    for (i, j) in all pairs do
        # Mask normalization (by the index i or j), then dilate the brain regions
        DB_i = BinaryDilate (B_i / i)
        DB_j = BinaryDilate (B_j / j)
        # Test of spatial contiguity
        if Sum (DB_i == DB_j) > 0) then
            # The ith and jth regions are neighbors (their dilated versions
             have non-vanishing overlap)
            Append (adjacency_list, (i, j))
    return adjacency_list
```

SI-4. Supporting Figures

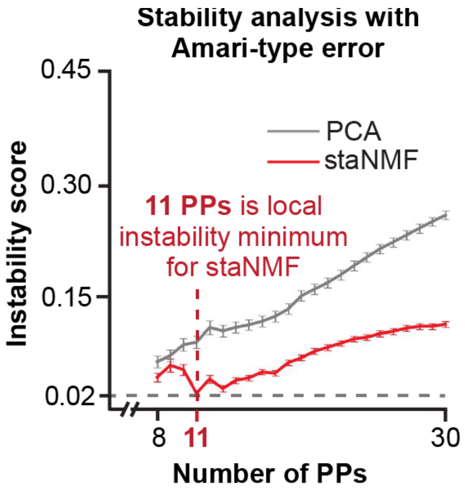

Figure S1: Stability analysis with Amari-type error function. Instability score of staNMF PPs and PCA PPs across 100 runs for each K value, from 8 to 30 for ABA dataset. The error bars are the standard deviation. This figure uses Amari-type error (4), while Fig. 1B uses the Hungarian matching method (5). Both approaches identify K = 11 for the minimum instability score (and thus most stability) for staNMF PPs.

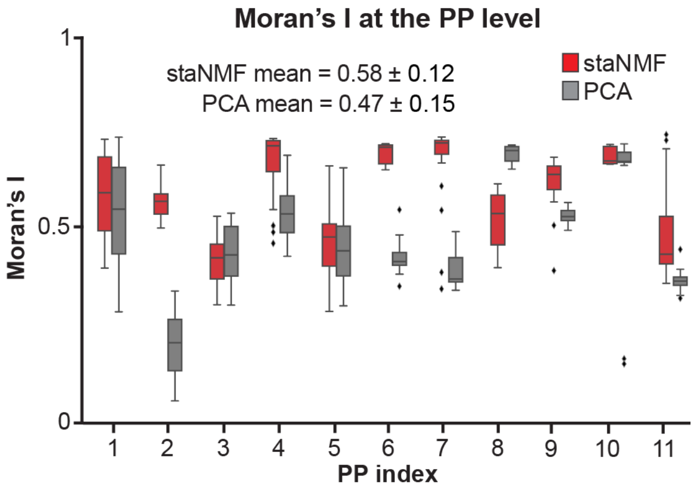

Figure S2: Moran's I per PP from staNMF and PCA. The plot uses data from 20 bootstrap simulations for each PP, for a total of 220 simulations for staNMF PPs and 220 simulations for PCA PPs. The mean Moran's I was 0.58 ± 0.12 for staNMF and 0.47 ± 0.15 for PCA. The p-value between the two samples was <0.001. The PPs from staNMF show greater spatial coherence, or higher Moran's I (1), than those from PCA for all but one case (PP8).

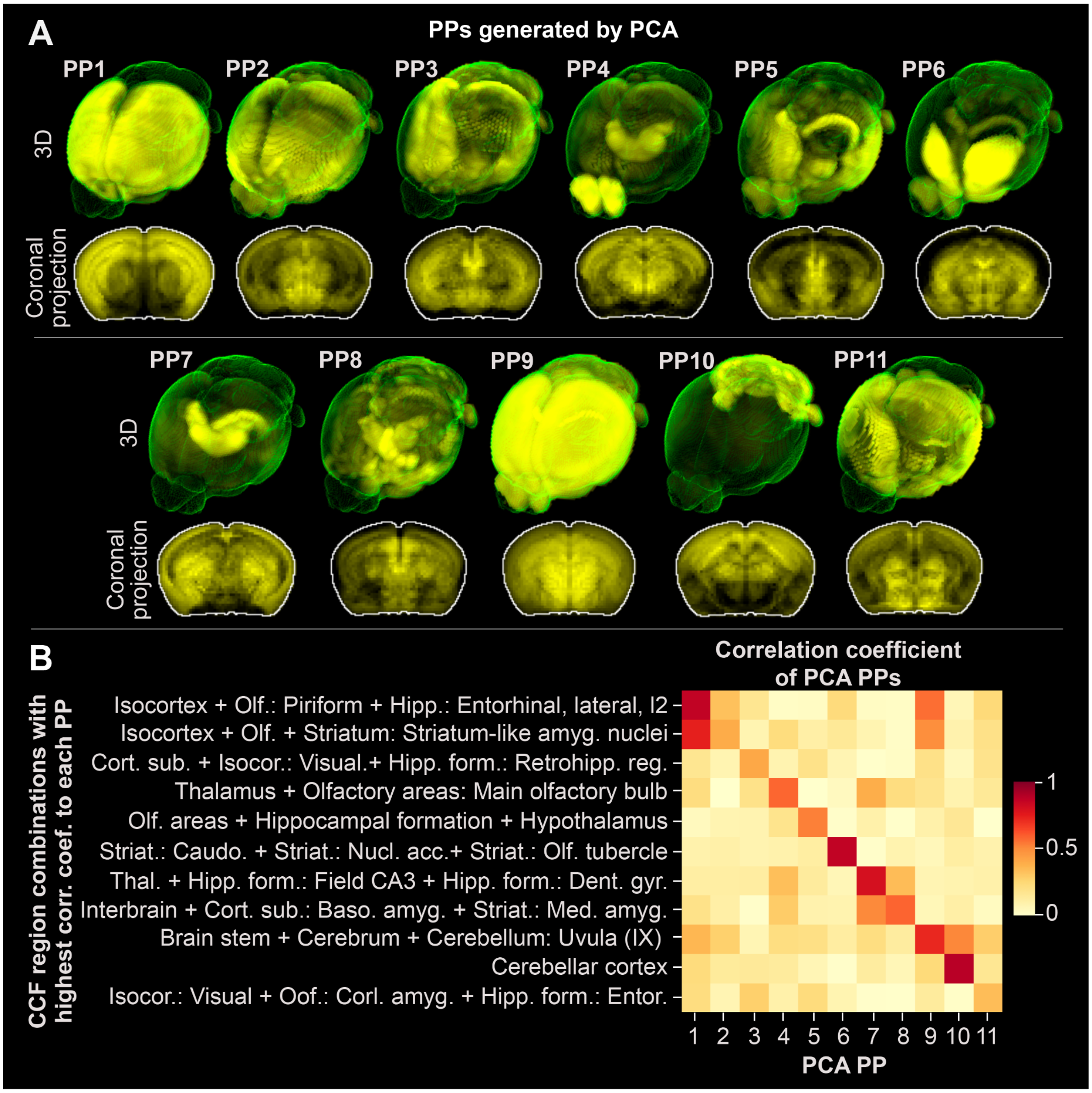

Figure S3: Similarity of PCA PPs to the expert-annotated brain regions. A. 11 PPs generated by PCA, ordered based on highest coarse region correlation to the CCFv3 ontology (6) in 3D and projected on the coronal plane. **B.** Heat map of the correlation coefficient between PCA PPs and the most similar combination of CCF regions (with the highest correlation coefficient).

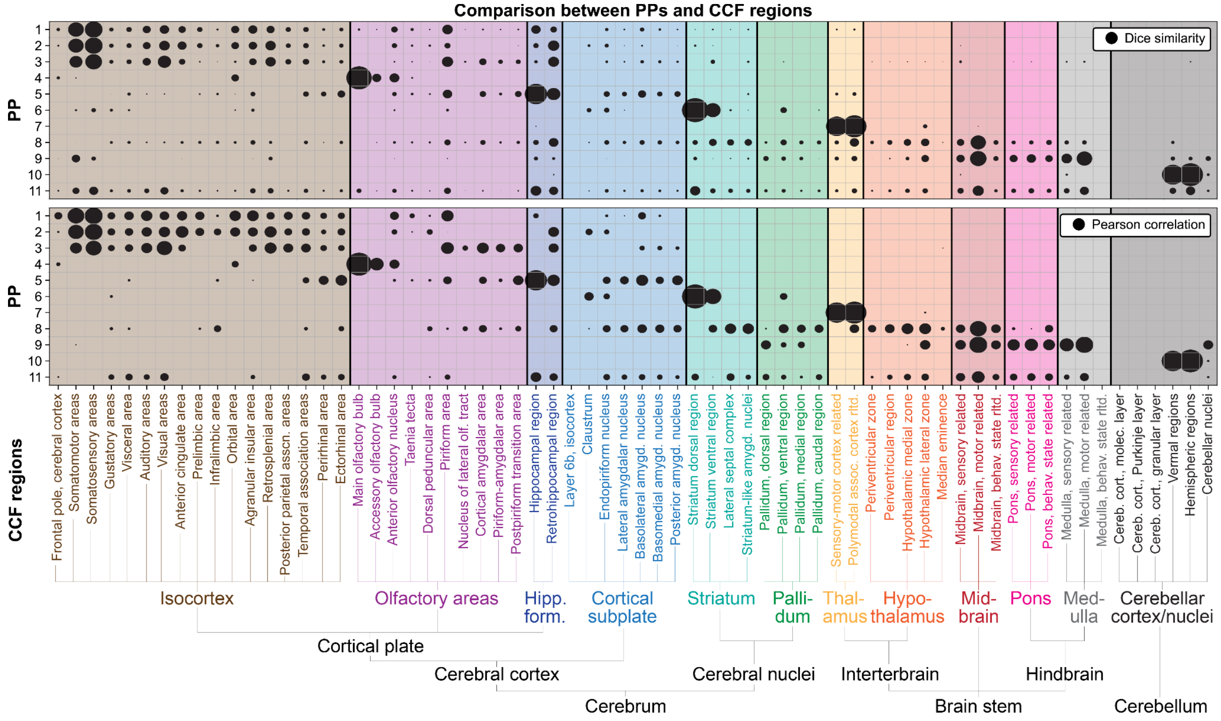

Figure S4: Metrics for region-level comparison between staNMF PPs and the CCF. The PPs from staNMF and CCF regions (6) are compared using the Dice similarity (top) and the Pearson correlation (bottom) visualized as the size of the filled circle. The PPs and CCF regions are arranged the same way as in Fig. 2 and Fig. S5.

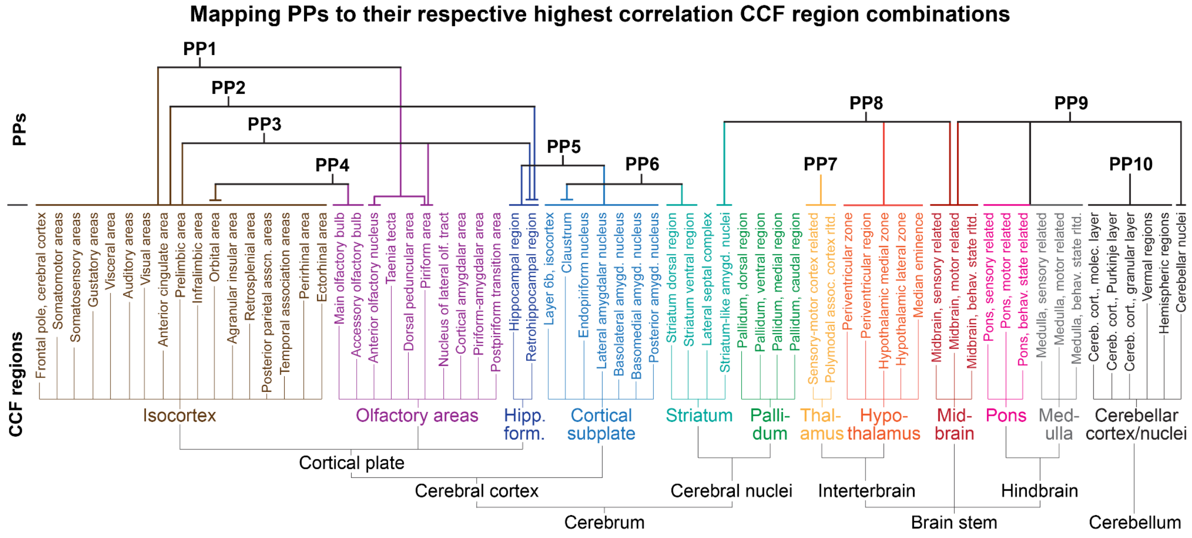

Figure S5: Summary of staNMF PPs linked to the best-fit combination of Allen CCF regions. The 10 PPs from main text Fig. 3 mapped to their best-fit combinations of CCF regions (6).

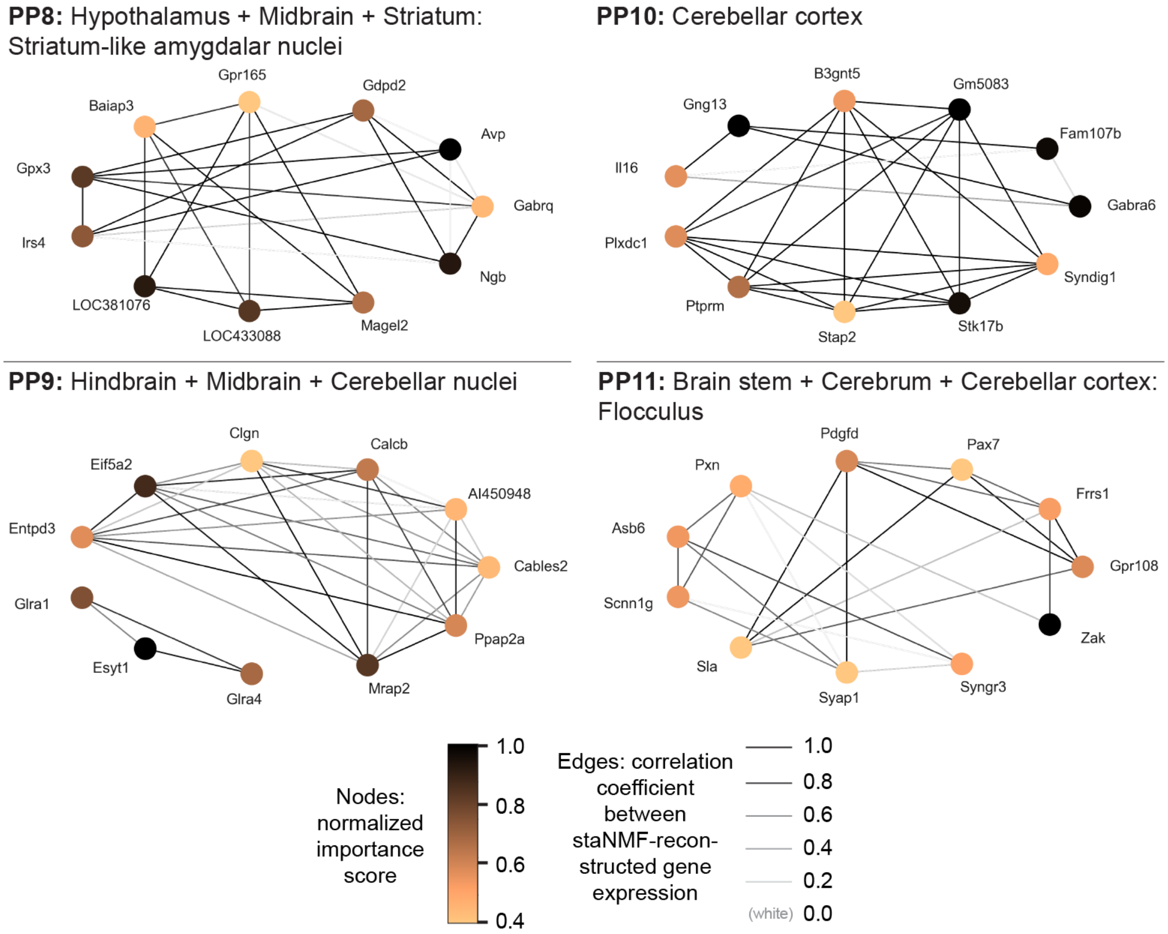

Figure S6: Putative spatial gene co-expression network construction, continued. Extension of Fig. 6 of the main text, the sGCNs from PPs 8-11 and their associated brain regions from the CCFv3 (6) are shown. The node color presents the selectivity of the gene to the PP associated with the brain region. An edge is drawn between genes if the similarity score is among the top 5% of all similarity scores for that gene subset. The edge color is proportional to the Pearson correlation of the reconstructed gene expression images of the two co-expressed genes.

Table S1: Region-specific marker genes from ISH and scRNA-seq data. Comparison of the PP-level (from ISH data) (7) and cell type-specific (from scRNA-seq data) (8) marker genes in the same spatial region of the adult mouse brain. The cell types subclass labels are from the Common Cell Type Nomenclature (CCN) (9).

PP index	Subclass of cell type in CCN	Marker gene
1	005 L5 IT CTX Glut	Arhgap25
1	034 NP PPP Glut	Rxfp1
1	274 PDTg Otp Shroom3 Gaba	Rxfp1
2	103 PVHd-DMH Lhx6 Gaba	Glra1
2	156 MB-ant-ve Dmrta2 Glut	Glra1
2	243 PGRN-PARN-MDRN Hoxb5 Glut	Sncg
2	261 HB Calcb Chol	Calcb
3	062 STR D2 Gaba	Adora2a
3	292 MV Nkx6-1 Gly-Gaba	Serpina9
4	072 LSX Sall3 Lmo1 Gaba	Ptprm
4	313 CBX Purkinje Gaba	Pcp2
4	314 CB Granule Glut	Gabra6
5	077 CEA-BST Gal Avp Gaba	Avp
5	107 DMH Hmx2 Gaba	Dlk1
5	108 ARH-PVp Tbx3 Gaba	Dlk1
5	222 PB Evx2 Glut	Gabrq
5	226 PRNc-PARN Tlx1 Glut	Dlk1
5	296 RPA Pax6 Hoxb5 Gly-Gaba	Dlk1
6	007 L2/3 IT CTX Glut	Stard8
6	009 L2/3 IT PIR-ENTl Glut	Igfn1
6	122 LHA-MEA Otp Glut	Lhx2
6	219 PB-SUT Tlx3 Lhx2 Glut	Lhx2
6	319 Astro-TE NN	Lhx2
8	016 CA1-ProS Glut	Spink8
8	037 DG Glut	Prox1
8	097 PVHd-SBPV Six3 Prox1 Gaba	Prox1
8	102 DMH-LHA Gsx1 Gaba	Prox1
8	147 AD Serpinb7 Glut	C1ql2
8	163 APN C1ql2 Glut	C1ql2
9	011 L2 IT ENT-po Glut	Lef1
9	107 DMH Hmx2 Gaba	Lef1
9	125 DMH Hmx2 Glut	Lef1
9	130 LHA Pmch Glut	Pmch
9	152 RE-Xi Nox4 Glut	Rgs16
9	187 SCsg Pde5a Glut	Lef1
9	205 SC-PAG Lef1 Emx2 Gaba	Lef1

9	206 SCm-PAG Cdh23 Gaba	Lef1
9	208 SC Lef1 Otx2 Gaba	Lef1
10	035 OB Eomes Ms4a15 Glut	Eomes
10	045 OB-STR-CTX Inh IMN	Dlx1
10	098 AHN-SBPV-PVHd Pdrm12 Gaba	Dlx1
10	105 TMd-DMH Foxd2 Gaba	Dlx1
10	107 DMH Hmx2 Gaba	Dlx1
10	260 MDRNv Crp Glut	Sp8
10	289 MDRNd Prox1 Pax6 Gly-Gaba	Sp8
11	137 PH-an Pitx2 Glut	Pax7
11	192 PPN-CUN-PCG Otp En1 Gaba	Pax7
11	204 SC Otx2 Gcnt4 Gaba	Pax7
11	205 SC-PAG Lef1 Emx2 Gaba	Pax7
11	208 SC Lef1 Otx2 Gaba	Pax7
11	209 SCs Pax7 Nfia Gaba	Pax7
11	212 SCs Lef1 Gli3 Gaba	Pax7
11	277 DTN-LDT-IPN Otp Pax3 Gaba	Pax7
11	280 NLL-po Pax7 Gaba	Pax7

References

1. Moran, P. A. P. Notes on Continuous Stochastic Phenomena. Biometrika 37, 17–23 (1950).
2. J. Hu, et al., SpaGCN: Integrating gene expression, spatial location and histology to identify spatial domains and spatially variable genes by graph convolutional network. Nat. Methods 18, 1342–1351 (2021).
3. Napari: Multi-dimensional image viewer for python. Information available at https://napari.org/stable/.
4. Amari, S., Cichocki, A. & Yang, H. A New Learning Algorithm for Blind Signal Separation. in Advances in Neural Information Processing Systems vol. 8 (MIT Press, 1995).
5. Kuhn, H. The Hungarian Method for the assignment problem. Naval Res Logist Q2, 83–97 (1955).
6. Wang, Q. et al. The Allen mouse brain common coordinate framework: a 3D reference atlas. Cell 181, 936–953 (2020).
7. Lein, E. S. et al. Genome-wide atlas of gene expression in the adult mouse brain. Nature 445, 168–176 (2007).
8. Z. Yao, et al., A high-resolution transcriptomic and spatial atlas of cell types in the whole mouse brain. Nature 624, 317–332 (2023).
9. J. A. Miller, et al., Common cell type nomenclature for the mammalian brain. eLife 9, e59928 (2020).
